# Prediction model for the onset risk of impaired fasting glucose: a 10-year longitudinal retrospective cohort health check-up study

**DOI:** 10.1186/s12902-021-00878-4

**Published:** 2021-10-22

**Authors:** Yuqi Wang, Liangxu Wang, Yanli Su, Li Zhong, Bin Peng

**Affiliations:** 1 Department of Epidemiology and Health Statistics, School of Public Health and Management, Chongqing Medical University, 400016 Chongqing, China; 2 Medical Data Research Institute of Chongqing Medical University, 400016 Chongqing, China; 3 School of Basic Medicine, Kunming Medical University, 650031 Kunming, China; 4 The First Affiliated Hospital of Chongqing Medical University Health Management Centre, 400016 Chongqing, China

**Keywords:** Impaired fasting glucose, Health check-up cohort, Prediction model

## Abstract

**Background:**

Impaired fasting glucose (IFG) is a prediabetic condition. Considering that the clinical symptoms of IFG are inconspicuous, these tend to be easily ignored by individuals, leading to conversion to diabetes mellitus (DM). In this study, we established a prediction model for the onset risk of IFG in the Chongqing health check-up population to provide a reference for prevention in a health check-up cohort.

**Methods:**

We conducted a retrospective longitudinal cohort study in Chongqing, China from January 2009 to December 2019. The qualified subjects were more than 20 years old and had more than two health check-ups. After following the inclusion and exclusion criteria, the cohort population was randomly divided into a training set and a test set at a ratio of 7:3. We first selected the predictor variables through the univariate generalized estimation equation (GEE), and then the training set was used to establish the IFG risk model based on multivariate GEE. Finally, the sensitivity, specificity, and receiver operating characteristic curves were used to verify the performance of the model.

**Results:**

A total of 4,926 subjects were included in this study, with an average of 3.87 check-up records, including 2,634 males and 2,292 females. There were 442 IFG cases during the follow-up period, including 286 men and 156 women. The incidence density was 26.88/1000 person-years for men and 18.53/1000 person-years for women (*P*<0.001). The predictor variables of our prediction model include male (relative risk (RR) =1.422, 95 % confidence interval (CI): 0.923-2.193, *P*=0.3849), age (RR=1.030, 95 %CI: 1.016-1.044, *P*<0.0001), waist circumference (RR=1.005, 95 %CI: 0.999-1.012, *P*=0.0975), systolic blood pressure (RR=1.004, 95 %CI: 0.993-1.016, *P*=0.4712), diastolic blood pressure (RR=1.023, 95 %CI: 1.005-1.041, *P*=0.0106), obesity (RR=1.797, 95 %CI: 1.126-2.867, *P*=0.0140), triglycerides (RR=1.107, 95 %CI: 0.943-1.299, *P*=0.2127), high-density lipoprotein cholesterol (RR=0.992, 95 %CI: 0.476-2.063, *P*=0.9818), low-density lipoprotein cholesterol (RR=1.793, 95 %CI: 1.085-2.963, *P*=0.0228), blood urea (RR=1.142, 95 %CI: 1.022-1.276, *P*=0.0192), serum uric acid (RR=1.004, 95 %CI: 1.002-1.005, *P*=0.0003), total cholesterol (RR=0.674, 95 %CI: 0.403-1.128, *P*=0.1331), and serum creatinine levels (RR=0.960, 95 %CI: 0.945-0.976, *P*<0.0001). The area under the receiver operating characteristic curve (AUC) in the training set was 0.740 (95 %CI: 0.712-0.768), and the AUC in the test set was 0.751 (95 %CI: 0.714-0.817).

**Conclusions:**

The prediction model for the onset risk of IFG had good predictive ability in the health check-up cohort.

## Background

Diabetes mellitus (DM) has become one of the most vital public health challenges faced by all countries in the 21st century and has become an epidemic in recent decades [1, 2]. Studies have shown that the global prevalence of DM was 8.5 % in 2014. It is estimated that the number of affected individuals will increase from 422 to 642 million by 2040 [3–5]. In China, despite the high incidence of DM, 50 % of patients are undiagnosed [6]. As early as 1997, the American Diabetes Association introduced the concept of impaired fasting glucose (IFG), which is a prediabetic condition [7–9]. IFG can be easily overlooked because of the unapparent clinical symptoms [10, 11], which makes it more desirable to have suitable risk assessment models to help individuals assess the risk of IFG. Therefore, the prediction model for the onset risk of IFG appears to be particularly important as an assessment tool. In recent years, both domestic and foreign researchers have developed many risk prediction models of DM, but there is a lack of risk prediction models of IFG for the health check-up cohort [12–14].

With the strengthening of health awareness, health check-ups have become an important method of health management [15, 16]. Health checkup data have accumulated comprehensive health information for many years, which is part of longitudinal data [17]. Owing to the nature of longitudinal data and the purpose of analysis, methods dedicated to longitudinal data analysis should be used [18]. Therefore, this study was based on the Chongqing health check-up longitudinal cohort to establish a prediction model using the generalized estimation equation (GEE) to assess the onset risk of IFG in the regular health check-up cohort and provide a reference for prevention in the health check-up cohort.

## Methods

### Study population

This retrospective, longitudinal cohort study began with a review of the health check-up records of 4,926 subjects (2,634 men and 2,292 women), whose ages ranged from 20 to 85 years, and who had more than two health check-ups at the Medical Examination Centre of the First Affiliated Hospital of Chongqing Medical University from January 2009 to December 2019. The annual health check-ups record included anthropometric measurements and the laboratory measurements. The inclusion criteria were as following: (1) no IFG or no DM or related diseases at baseline; (2) at least two health check-up records and complete physical check-up data; and (3) age ≥20 years. The exclusion criteria were as follows:(1) had IFG or DM or related diseases at baseline; (2) were taking hypoglycemic drugs; and (3) were lost to the follow-up and lacked key information. The cohort observation time was the period from the first health checkup to the onset of IFG (6.1≤IFG≤6.9) or the last health checkup without IFG. The outcome of this study was the presence of IFG; that is, once the subjects had IFG, the subsequent data were not included in the study.

**Fig. 1:**
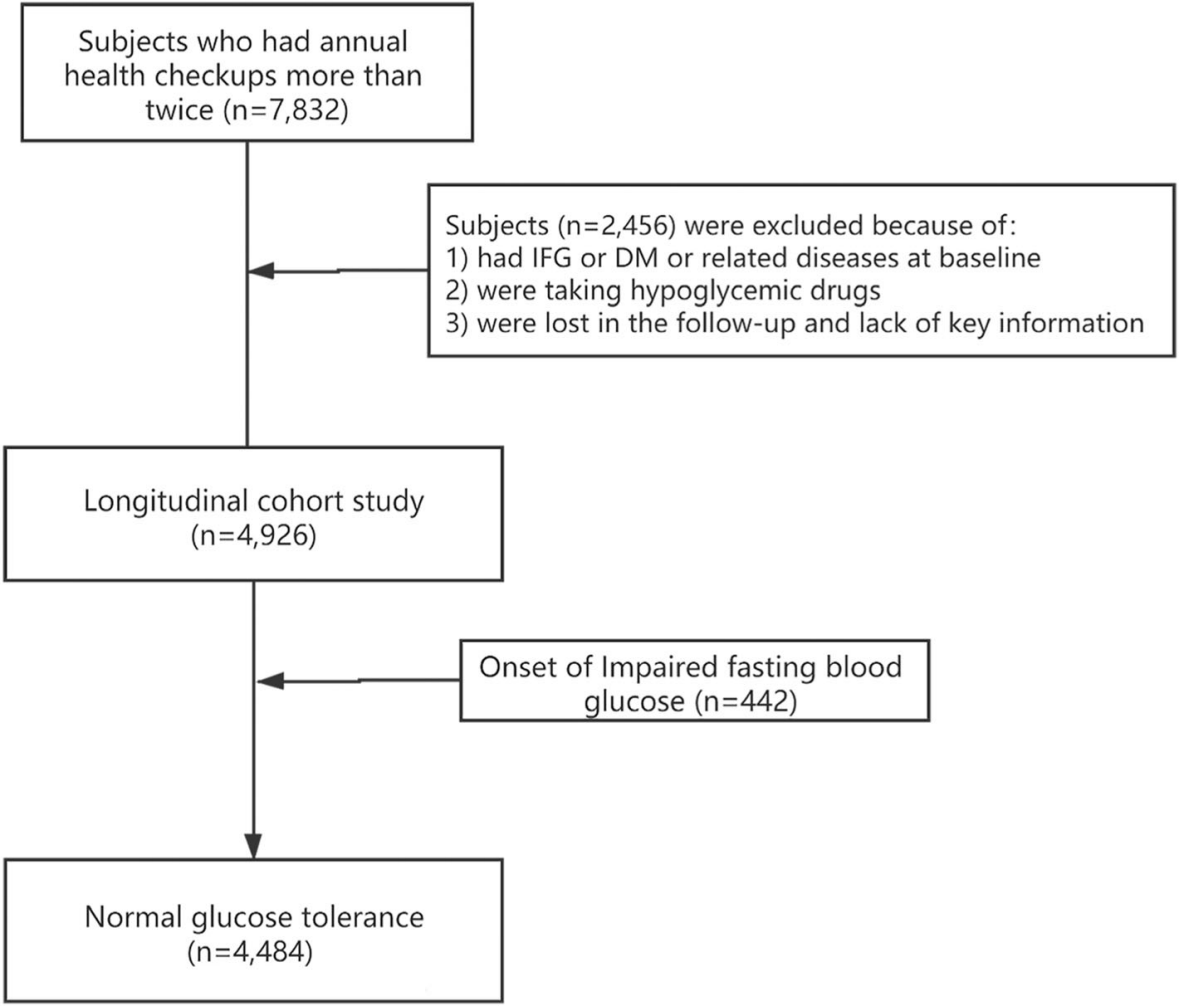
Flow diagram of the study design and participant selection process for the study

During the check-up period at a certain university, the person in charge of the check-up at the university will go to the Medical Examination Centre of the First Affiliated Hospital of Chongqing Medical University. witnessed by the person in charge of the university, the researchers of the medical examination center will verbally inform the participants about using the check-up records for scientific research in the future, in order to analyse the influencing factors of chronic diseases in Chongqing. The participants agreed and provided verbal informed consent. All participants were assigned a numeric code that was used throughout the study, and all data were stored in a secure database to maintain anonymity. The selection process for the participants in this study is shown in Fig. 1.

### Measurements

The health check-up was performed at the Medical Examination Centre of the First Affiliated Hospital of Chongqing Medical University, which has obtained ISO-15,189 standard certification. The health checkup included the following:

#### Anthropometric measurements

Anthropometric measurements were performed with the subjects wearing light clothes. Waist circumference (WC) was measured with a soft ruler with a minimum scale of 0.1 cm. The measurer stood in front of the measured person, wrapped the measuring tape around the waist horizontally along the measuring point, repeated the measurement twice and recorded the average value [19]. Weight and height were measured in a standing position using calibrated weighing scales, and body mass index (BMI) was calculated as weight (*kg*) divided by height (*m*) squared. After the subjects sat for at least 5 min, a HEM-906 sphygmomanometer (Omron Matsuzak Co., Ltd., Japan) was used to measure the blood pressure of the right upper limb of the subjects. Systolic blood pressure (SBP) and diastolic blood pressure (DBP) were measured for 3 consecutive times with an interval of 30 s, and the average value of the three measurements was taken as the blood pressure value.

#### Laboratory measurements (baseline and follow-up)

Fasting venous blood samples (5 mL) were collected from the antecubital vein after an overnight fasting and stored at -20 °C until analysis. Fasting blood glucose (FBG), total cholesterol (TC), triglycerides (TG), low-density lipoprotein cholesterol (LDL-C), high-density lipoprotein cholesterol (HDL-C), serum creatinine (SCr), serum uric acid (SUA), and blood urea levels were measured using an automatic biochemical analyser (Hitachi 7020; Tokyo, Japan).

### Model outcome: IFG definition

According to the Chinese Type II Diabetes Prevention and Control Guidelines (2017 Edition), an FBG between 3.9 and 6.0 mmol/L was consider non-IFG, and FBG between 6.1 and 6.9 mmol/L was consider as IFG [20].

### Model predictor variables

Pre-specified predictor variables were analysed, based on prior evidence and availability: age [21], sex [21], BMI [22] (According to the World Health Organization (WHO) criteria, BMI were categorized into four groups as underweight [BMI≤18.5 kg/m²], normal [18.5≤BMI≤23.9 kg/m²], overweight [24.0≤BMI≤27.9 kg/m²], obesity [BMI≥28 kg/m²]), WC [23], SBP [23], DBP [23], four items of blood lipids [24] (TC, TG, LDL-C, and HDL-C), three items of renal function [25] (SCr, SUA, and blood urea).

### Statistical analysis

A descriptive analysis of the baseline characteristics was performed. Continuous variables were analysed by *Student’s t-test*, which was expressed as mean ± standard deviation (SD); the categorical variables were tested using the *chi-squared* test. The incidence density was estimated based on the number of new cases and the number of years of observation, and the trend of IFG incidence density with age was analysed using the *Cochran-Armitage* trend test.

We randomly selected 70 % of the cohort of subjects as the training set, and the remaining 30 % as the test set. We first selected the predictor variables through univariate GEE (*P*<0.20), then used multivariate GEE to establish the prediction model for the onset risk of IFG through the training set, and finally used the test set to verify the performance of the established model through sensitivity, specificity, and the under receiver operating characteristic curve (AUC). The GEE was implemented by the GENMOD module in SAS9.4, and all data were analysed using SAS9.4 statistical software (version 9.4; SAS Institute Inc., Cary, North Carolina).

## Results

### Study cohort

A total of 4,926 subjects with an average age of 44.85±14.80 years were included in the cohort, including 2,634 men and 2,292 women. Subjects in the cohort participated in the check-up at most nine times and at least two times, with an average of 3.87 times. The follow-up results for different sex and ages are summarized in Table 1. During the follow-up period, 442 new cases of IFG were diagnosed, including 286 men and 156 women. The total incidence density was 23.21/1000 person-years, male incidence density was 26.88/1000 person-years, female incidence density was 18.53/1000 person-years, and males were higher than females (*P*<0.0001). The *Cochran Armitage* test found that there was a linear trend between IFG incidence density and age, and the incidence density showed an obvious increasing trend with increasing of age (Z=-12.5907, *P*<0.0001).

**Table 1:** IFG incidence density of different gender and age

Group	Number of cases	Incidence density (/1000 person-years)	*P*
Sex			<0.0001
Female	156	18.53	
Male	286	26.88	
Age			<0.0001*
20~	18	8.33	
30~	61	9.91	
40~	113	23.26	
50~	94	35.58	
60~	90	50.99	
70~	66	45.08	
Total	442	23.21	

Note: *this probability value is the probability value of the trend

### Baseline characteristics

Table 2 shows that at baseline, the average age of the IFG group was (53.37±14.26) years, and the average age of the non-IFG group was (43.99±14.59) years. The difference was statistically significant. When entering the cohort, the BMI of the IFG group was significantly higher than that of the non-IFG group; the SBP, DBP, TC, TG, LDL-C, blood urea, SCr, and SUA of the IFG group were higher than that of the non-IFG group, while HDL-C was lower than the non-IFG group. Compared with IFG group, except height, there were significant differences in age, weight, BMI, WC, SBP, DBP, TC, TG, HDL-C, LDL-C, blood urea, SCr and SUA between the two groups (*P*<0.05).

**Table 2:** Baseline characteristics of the cohort population with IFG and non-IFG

Variable	IFG (*N* =442)	Non-IFG(*N* =4,484)	*P*-value
Age	53.37±14.26	43.99±14.59	<0.0001
Sex			<0.0001
Male	286	2,348	
Female	156	2,136	
Height	163.70±8.39	163.80±8.21	0.7064
Weight	66.02±11.24	61.89±11.15	<0.0001
BMI	24.54±3.06	22.95±2.99	<0.0001
WC	83.29±8.74	78.70±9.07	<0.0001
SBP	131.30±19.78	120.50±17.21	<0.0001
DBP	79.00±11.75	73.84±10.97	<0.0001
TC	5.02±0.97	4.75±0.90	<0.0001
TG	1.88±1.58	1.38±0.96	<0.0001
HDL-C	1.39±0.37	1.46±0.35	<0.0001
LDL-C	3.02±0.82	2.84±0.80	<0.0001
Blood urea	5.37±1.33	5.03±1.33	<0.0001
SCr	73.87±14.99	70.29±16.27	<0.0001
SUA	352.90±80.42	327.60±86.09	<0.0001

Abbr: *IFG* Impaired fasting glucose, *BMI* Body mass index, *WC* Waist circumference, *SBP* Systolic Blood pressure, *DBP* Diastolic Blood pressure, *TC* Total cholesterol, *TG* Triglycerides, *HDL-C* High-density lipoprotein cholesterol, *LDL-C* Low-density lipoprotein cholesterol, *SCr* Serum creatinine, *SUA* Serum uric acid

### Establishment of the prediction model for the onset risk of IFG

Taking IFG as the dependent variable, the sex, age, BMI, WC, SBP, DBP, TC, TG, HDL-C, LDL-C, blood urea, SCr, and SUA of the subjects were used as independent variables to fit the GEE. As shown in Table 3, we selected all possible predictor variables from univariate GEE (*P*<0.20). We established a prediction model for the onset risk of IFG by inputting the possible predictor variables. Table 4 shows the predictors included sex, age, BMI, WC, SBP, DBP, TC, TG, HDL-C, LDL-C, blood urea, SCr, and SUA. Male (RR =1.422, 95 %CI: 0.923-2.193, *P*=0.3849), age (RR =1.030, 95 %CI: 1.016-1.044, *P*<0.0001), WC (RR =1.005, 95 %CI: 0.999~1.012, *P*=0.0975), SBP (RR =1.004, 95 %CI: 0.993~1.016, *P*=0.4712), DBP (RR =1.023, 95 %CI: 1.005-1.041, *P*=0.0106), Obesity (RR=1.797, 95 %CI: 1.126-2.867, *P*=0.0140), TG (RR=1.107, 95 %CI: 0.943-1.299, *P*=0.2127), HDL-C (RR=0.992, 95 %CI: 0.476-2.063, *P*=0.9818), LDL-C (RR=1.793, 95 %CI: 1.085-2.963, *P*=0.0228), blood urea (RR=1.142, 95 %CI: 1.022-1.276, *P*=0.0192) and SUA (RR=1.004, 95 %CI: 1.002-1.005, *P*=0.0003) were risk factors. Conversely, TC (RR=0.674, 95 %CI: 0.403-1.128, *P*=0.1331), HDL-C (RR=0.992, 95 %CI: 0.476-2.063, *P*=0.9818) and SCr (RR=0.960, 95 %CI: 0.945-0.976, *P*<0.0001) were protective factors. According to the coefficients obtained by multivariate GEE, a prediction model for the onset of IFG can be obtained.

**Table 3:** Univariate GEE analysis results

Variable	$\beta$	RR	95 %CI	*P*-value
Male	0.379	1.363	(1.202, 1.774)	0.0001
Age	0.034	1.035	(1.028, 1.041)	<0.0001
BMI				
Underweight	-0.373	0.689	(0.364, 1.303)	0.2517
Overweight	0.613	1.846	(1.504, 2.263)	<0.0001
Obesity	1.139	3.124	(2.317, 4.208)	<0.0001
WC	0.009	1.009	(1.001, 1.019)	0.0429
SBP	0.043	1.044	(1.023, 1.033)	<0.0001
DBP	0.037	1.038	(1.029, 1.046)	<0.0001
TC	0.324	1.383	(1.249, 1.530)	<0.0001
TG	0.120	1.127	(1.067, 1.191)	<0.0001
HDL-C	-0.646	0.524	(0.384, 0.715)	<0.0001
LDL-C	0.383	1.467	(1.312, 1.640)	<0.0001
Blood urea	0.148	1.160	(1.095, 1.228)	<0.0001
SCr	0.003	1.003	(0.999, 1.007)	0.1791
SUA	0.004	1.004	(1.003, 1.004)	<0.0001

Note: The sex was female as the reference group, BMI was normal as the reference group, and RR was 1.0

**Table 4:** Multivariate GEE analysis results

Variable	$\beta$	RR	95 %CI	*P*-value
Male	0.3523	1.422	(0.923,2.193)	0.3849
Age	0.0291	1.030	(1.016,1.044)	<0.0001
WC	0.0053	1.005	(0.999,1.012)	0.0975
SBP	0.0042	1.004	(0.993,1.016)	0.4712
DBP	0.0230	1.023	(1.005,1.041)	0.0106
BMI				
Underweight	0.2112	1.235	(0.494,3.090)	0.6516
Overweight	0.1924	1.212	(0.869,1.690)	0.2569
Obesity	0.5860	1.797	(1.126,2.867)	0.0140
TC	-0.3948	0.674	(0.403,1.128)	0.1331
TG	0.1018	1.107	(0.943,1.299)	0.2127
HDL-C	-0.0085	0.992	(0.476,2.063)	0.9818
LDL-C	0.5836	1.793	(1.085,2.963)	0.0228
Blood urea	0.1328	1.142	(1.022,1.276)	0.0192
SCr	-0.0404	0.960	(0.945,0.976)	<0.0001
SUA	0.0035	1.004	(1.002,1.005)	0.0003

Note: the gender was female as reference group, BMI was normal as reference group, RR was 1.0. According to the parameters listed in Table 4, we can obtain a formula to compute LogitP of IFG

### The performance of prediction model for the onset risk of IFG

**Fig. 2:**
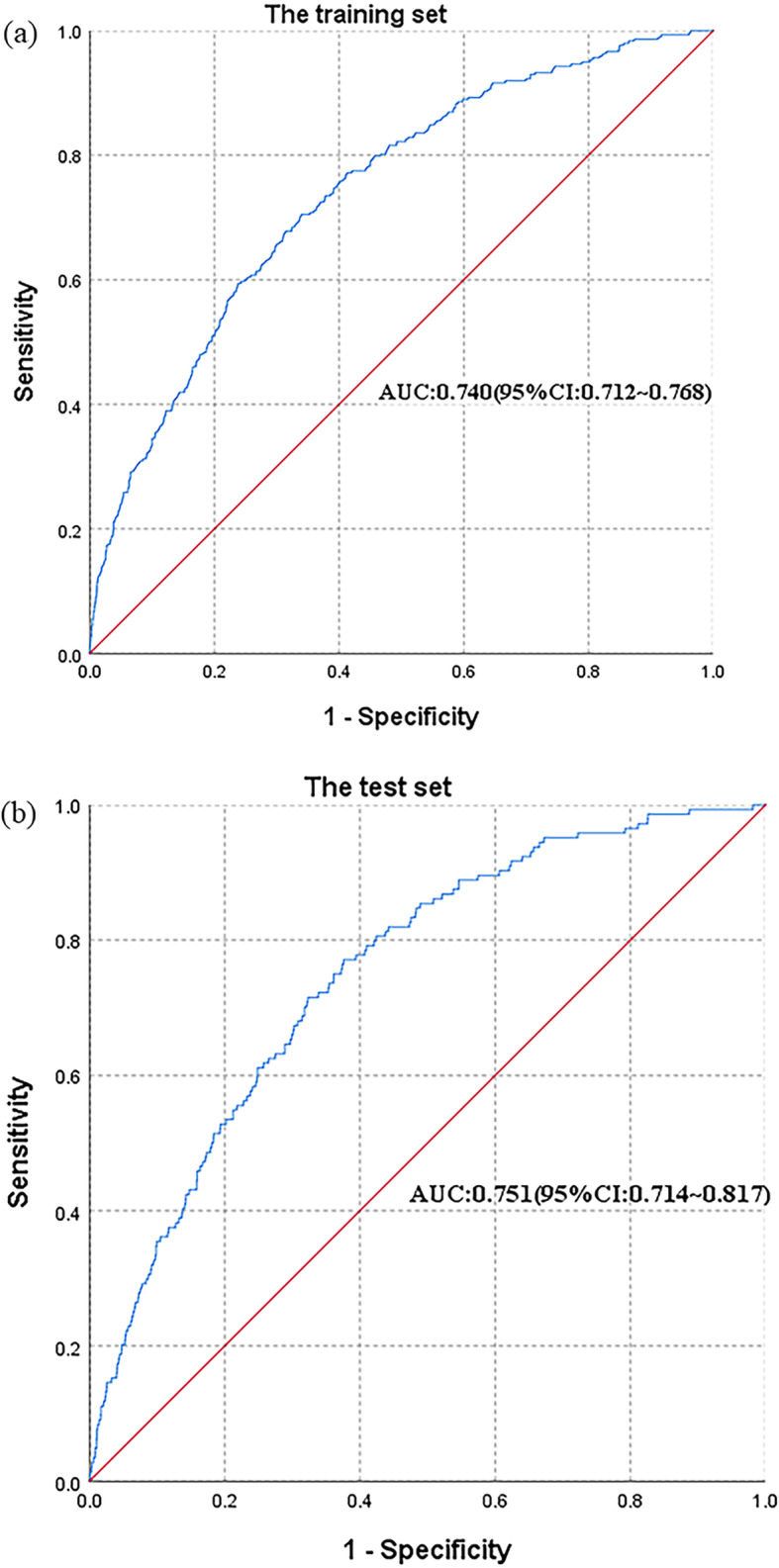
The receiver operating characteristic (ROC) curves of prediction model for the onset risk of IFG. **A** for the training set and (**B**) for the test set

Figure 2 summarizes the area under the receiver operating characteristic curve (AUC) obtained from the training and test sets of the prediction model for the onset risk of IFG. Figure 2 shows the AUC obtained from the training set and test set of the prediction model for the onset risk of IFG, and judges the discriminant ability of the prediction model based on the AUC. The AUC of the training set was 0.740 (95 %CI, 0.712-0.768), and the AUC of the test set was 0.751 (95 %CI, 0.714-0.817). Therefore, the prediction model for the onset risk of IFG had good discrimination ability and good predictive ability in the health check-up cohort. The best cut-off point was 2.12 %, and the sensitivity and specificity of the training and test sets were 70.5 % (71.5 %) and 66.1 % (66.8 %), respectively.

## Discussion

IFG was defined FBG level is higher than normal, but not high enough to diagnose DM, and belongs to the prediabetic condition [7, 26]. The survey results revealed that the national rate of IFG in middle-aged and elderly people was 7.3 %, which increased to 9.7 % in a 2018 study [27, 28]. Studies have shown that the IFG is an obligatory process from a healthy state to DM. If IFG was detected and immediate measures were taken, the progress of DM may have been delayed or halted. If it was not controlled, it can easily lead to DM [29]. The results of Yeboah et al. [30] indicated that IFG is an independent risk factor for the development of type II DM, which means that interventions to reduce the incidence of IFG will ultimately reduce the incidence of type II DM. Early diagnosis and intervention of IFG are effective in controlling the incidence of DM [31].

IFG can not only prevent the occurrence of DM, but it is also related to the occurrence of cardiovascular disease (CVD). Studies have shown that patients with IFG have a significantly increased CVD risk compared to patients with normal glucose tolerance [32]. A study used a national epidemiological database to explore the different types of FBG (normal, normal-high, IFG, and DM) and the risk of future CVD (including coronary heart disease, stroke, heart failure, etc.) in young adults. The results showed that the risk of CVD increased with an increase in FBG levels, and the risk of myocardial infarction in the IFG category began to increase significantly [33]. Therefore, early identification of IFG plays an important role in the primary prevention of CVD. In 2010, Volpe et al. [34] proposed that understanding the relationship between abnormalities in blood glucose metabolism (or dysglycaemia) and CVD complications was a key point in the primary prevention of CVD.

Disease risk assessment is a key technique for chronic disease management and is an effective auxiliary diagnostic tool to identify high-risk groups [35]. In general, the development of risk prediction models incorporates general patient characteristics, clinical trial results and other data [36, 37]. At present, there is a lack of a risk assessment model for IFG in China. Except for a few studies that only focused on the epidemiological factors of IFG, there was no intuitive risk assessment model to predict IFG. In addition, most of the currently reported studies were cross-sectional studies. In this study, we conducted a longitudinal retrospective cohort study, including up to 10 years of health check-up dynamic change indicators, to predict the risk of IFG. We established a prediction model for the onset risk of IFG based on longitudinal physical check-up data and identified the predictor variables of the model through the GEE. Finally, the performance of the model was verified.

In recent years, health check-ups have become an important method of health management. The predictor variables of our risk model were readily available on health check-ups, including age, sex, BMI, WC, DBP, SBP, four4 items of blood lipids (TC, TG, LDL-C, and HDL-C), three of renal function (SCr, SUA, and blood urea), and others. SCr and TC were found to be protective factors for IFG by multivariate GEE (RR=0.960, 95 % CI: 0.945~0.976; RR=0.674, 95 % CI: 0.403~1.128, respectively). Yoshida et al. involved 7,905 participants in a community-based longitudinal cohort health examination study after adjusting for age, BMI, SBP, and metabolic disease-related variables. This study concluded that the level of SCr is related to the onset of IFG, and the lower the level of SCr, the more likely it is to lead to the development of IFG [25]. SCr acts as a protective factor against IFG, which may be associated with skeletal muscle mass. Skeletal muscle is the main target organ for the control of blood glucose, which produces SCr at a relatively constant rate after creatine and phosphocreatine metabolism [38, 39], and SCr is a measure of skeletal muscle mass [40]. When the SCr level is low, skeletal muscle capacity is low, implying fewer insulin targets, which explains why a lower SCr level causes IFG [41]. Hyperuricemia was defined as SUA> 416 mmol/L in men and 357 mmol/L in women [23, 24]. In our established model, hyperuricemia acted as an independent risk factor for predicting IFG, which was similar to the results of many studies and different from the results of Taniguchi et al., who did not find an association between SUA levels and the risk of type II DM through a retrospective cohort study [42–45]. Studies have shown that SUA interacts with the FBG levels. Hyperinsulinemia elevated SUA levels by reducing the excretion of SUA and the accumulation of SUA products, and an increase in SUA also decreased glucose uptake by insulin [46, 47]. We evaluated the performance of the model based on the sensitivity, specificity, and AUC. Sensitivity and specificity represent the ability of the model to identify positive and negative results, respectively. Generally, good quality prediction models have both high sensitivity and specificity [48]. Our risk prediction model could be used to screen undiagnosed individuals with IFG, because of its good sensitivity and specificity. The sensitivity and specificity of the training set were70.5 % and 66.1 %, respectively, and the sensitivity and specificity of the test set were 71.5 % and 66.8 % respectively. The best cut-off point in the model was 2.12 %, that is, when the probability was greater than 2.12 %, IFG occurred. The prediction accuracy of the model was evaluated by the magnitude of the AUC; the more accurate the prediction model, the greater the AUC. In general, an AUC greater than 0.7 can be considered good for the model predictive ability. For the established model, the AUCs of the training and the test sets were 0.740 and 0.751 respectively. The sensitivity, specificity, and AUC showed that the prediction model for the onset risk of IFG was valuable.

The advantages of this study include a large sample size and a long cohort time. In addition, all qualified participants underwent a complete health check-up. However, it should be noted that the present study had some limitations. First, we were only able to collect data annually; therefore, data collection was not truly continuous. Second, we investigated the health check-up cohort, which may limit the generalisation of our results to other populations. Finally, this study was a retrospective cohort study, and the lifestyle of the subjects was not investigated. Because many diseases are often closely related to lifestyle, a comprehensive study should be conducted in future research. We should not be limited to health check-up data, but should incorporate lifestyle and so on.

## Conclusions

The predictive ability of the risk model based on longitudinal health check-up data in the training and test sets was reliable, with simple predictor variables and risk forms. This model can help individuals assess the risk of IFG, and provide evidence for the primary prevention and control of DM and CVD.

## Data Availability

The datasets used and/or analyzed during the current study, are available from the corresponding author on reasonable request.

## Abbreviations

IFG: Impaired fasting glucose
GEE: Generalized estimation equation
BMI: Body mass index
DBP: Diastolic Blood pressure
SBP: Systolic Blood pressure
WC: Waist circumference
SD: Standard deviation
RR (95% CI): Relative risk with 95 % Confidence interval
AUCs: Area under receiver operating curves
TC: Total cholesterol
TG: Triglycerides
LDL-C: Low-density lipoprotein cholesterol
HDL-C: High-density lipoprotein cholesterol
SCr: Serum creatinine, SUA:Serum uric acid

## References

[1] Mirzaei M, Rahmaninan M, Mirzaei M, Nadjarzadeh A, tafti A: Epidemiology of diabetes mellitus, pre-diabetes, undiagnosed and uncontrolled diabetes in Central Iran: results from Yazd health study. BMC Public Health 2020, 20. 10.1186/s12889-020-8267-y PMC6998152 32013917

[2] Fan W (2017). Epidemiology in diabetes mellitus and cardiovascular disease. Cardiovascular Endocrinology.

[3] World Health O (2016). Global report on diabetes.

[4] Al-Lawati J (2017). Diabetes Mellitus: A Local and Global Public Health Emergency!. Oman Medical Journal.

[5] Pǎtru D, Mitrea A, Manea M, Preda SD, Mota M, Lacatis D: Diabetes mellitus epidemiology. 2011, 18:67-72.

[6] Hu JI L, Zhang S (2013). Challenge the huge economic burden that diabetes has brought to China with new strategies and management methods. China Medicine and Pharmacy.

[7] Mengzi S, Min W, Chong S, Pingping Z, Yaogai L, Liyuan P, Shuo L, Yan Y, Lina J: The cut-off value of impaired fasting glucose should be lower: Based on the associations of fasting blood glucose with blood lipids. Primary Care Diabetes 2019, 14. 10.1016/j.pcd.2019.07.004 31405610

[8] Hanefeld M, Temelkova-Kurktschiev T, Schaper F, Henkel E, Siegert G, Köhler C (1999). Impaired fasting glucose is not a risk factor for atherosclerosis. Diabetic medicine: a journal of the British Diabetic Association.

[9] Rondanelli M, Riva A, Petrangolini G, Allegrini P, Bernardinelli L, Fazia T, Peroni G, Gasparri C, Nichetti M, Faliva MA et al: The Metabolic Effects of Cynara Supplementation in Overweight and Obese Class I Subjects with Newly Detected Impaired Fasting Glycemia: A Double-Blind, Placebo-Controlled, Randomized Clinical Trial. Nutrients 2020, 12(11). 10.3390/nu12113298 PMC7693737 33126534

[10] Jacqueline MDekke, Zhang Y, Tan Y (2008). : Blue: Oppose the American Diabetes Association’s new standard for impaired fasting glucose. Clinical Journal of Diabetes World.

[11] Yang X, He Q, Zhou R, Peng Q, Xiong J, Zhang R (2016). Prevalence and risk factors of impaired fasting glucose among physical examination population in the western new city of Chongqing. Journal of Chongqing Medical University.

[12] Chien K-L, Cai T, Hsu H, Su T-C, Chang W-T, Chen M, Lee Y, Hu F (2009). A prediction model for type 2 diabetes risk among Chinese people. Diabetologia.

[13] Rathmann W, Kowall B, Heier M, Herder C, Holle R, Thorand B, Strassburger K, Peters A, Wichmann HE, Giani G (2010). Prediction models for incident Type 2 diabetes mellitus in the older population: KORA S4/F4 cohort study. Diabetic Medicine.

[14] Xiong X-l, Zhang R-x, Bi Y, Zhou W-h, Yu Y, Zhu D-l (2019). Machine Learning Models in Type 2 Diabetes Risk Prediction: Results from a Cross-sectional Retrospective Study in Chinese Adults. Current Medical Science.

[15] Zhang J, Li Q, Liu F, Han Y, Yang J (2019). Trend of health management services model. Journal of Shandong University(Health Sciences).

[16] Wen N, Liu Y, Yang H, Guan X, Shuai P, Wan Q (2020). Discussing The Key Points and Implementation Methods of Integrated Health Management Service Mode in Hospital Physical Examination Center. Chinese Health Service Management.

[17] Shi F: Research on common chronic disease risk factors measuring and risk rating appraisal. Ph.D. Fourth Military Medical University 2015.

[18] Zhang Q: Stduy on design and statistical analysis strategies for large sample longitudinal health management cohort data. Master.Shang dong university 2013.

[19] Wang L:Health Manager: National Vocational Qualification Level III: Health Manager: National Vocational Qualification Level III 2013.

[20] Chinese guidelines for the prevention and treatment of type 2 diabetes (2017 Edition), Chinese Journal of practical internal medicine 2018, 38 (04): 292-344

[21] Williams J, Zimmet P, Shaw J, de Courten M, Cameron A, Chitson P, Tuomilehto J, Alberti G (2003). Gender differences in the prevalence of impaired fasting glycaemia and impaired glucose tolerance in Mauritius. Does sex matter?. Diabetic medicine: a journal of the British Diabetic Association.

[22] Zhao Y, Liu Y, Sun H, Sun X, Yin Z, Li H, Ren Y, Wang B, Zhang D, Liu X (2018). Body mass index and risk of all-cause mortality with normoglycemia, impaired fasting glucose and prevalent diabetes: Results from the Rural Chinese Cohort Study. Journal of Epidemiology and Community Health.

[23] Gautier A, Roussel R, Ducluzeau P, Lange C, Vol S, Balkau B, Bonnet F (2010). Increases in Waist Circumference and Weight As Predictors of Type 2 Diabetes in Individuals With Impaired Fasting Glucose: Influence of Baseline BMI Data from the DESIR study. Diabetes care.

[24] Noale M, Maggi S, Zanoni S, Limongi F, Zambon S, Crepaldi G: Lipid risk factors among elderly with normal fasting glucose, impaired fasting glucose and type 2 diabetes mellitus. The Italian longitudinal study on aging. Nutrition, metabolism, and cardiovascular diseases: NMCD 2011, 23 10.1016/j.numecd.2011.06.004 21937208

[25] Yoshida N, Miyake T, Yamamoto S, Furukawa S, Senba H, Kanzaki S, Koizumi M, Ishihara T, Yoshida O, Hirooka M (2019). The Serum Creatinine Level Might Be Associated with the Onset of Impaired Fasting Glucose: A Community-based Longitudinal Cohort Health Checkup Study. Internal Medicine.

[26] Alberti G, Zimmet PZ: Definition, diagnosis and classification of diabetes mellitus and its complications. Part 1 Diagnosis and classification of diabetes mellitus Provisional report of a WHO consultation Diabet Med 1998, 15. 10.1002/(SICI)1096-9136(199807)15:7<539::AID-DIA668>3.0.CO;2-S 9686693

[27] Gu D, Reynolds K, Duan XF, An X, Chen J, Wu XG, Mo JP, Whelton P, He J (2003). Erratum to: Prevalence of diabetes and impaired fasting glucose in the Chinese adult population: International Collaborative Study of Cardiovascular Disease in Asia (InterASIA). Diabetologia.

[28] Cho NH, Shaw J, Karuranga S, Huang Y, da Rocha Fernandes JD, Ohlrogge A, Malanda B: IDF Diabetes Atlas: Global estimates of diabetes prevalence for 2017 and projections for 2045. Diabetes Research and Clinical Practice 2018, 138. 10.1016/j.diabres.2018.02.023 29496507

[29] Huang Y: IDF Diabetes Atlas 8th Edition; 2017.

[30] Yeboah J, Bertoni A, Herrington D, Post W, Burke G (2011). Impaired Fasting Glucose and the Risk of Incident Diabetes Mellitus and Cardiovascular Events in an Adult Population MESA (Multi-Ethnic Study of Atherosclerosis). Journal of the American College of Cardiology.

[31] Gerstein H, Santaguida P, Raina P, Morrison K, Balion C, Hunt D, Yazdi H, Booker L (2008). Annual incidence and relative risk of diabetes in people with various categories of dysglycemia: A systematic overview and meta-analysis of prospective studies. Diabetes research and clinical practice.

[32] Danaei G, Lawes C, Vander Hoorn S, Murray C, Ezzati M (2006). Global and regional mortality from ischaemic heart disease and stroke attributable to higher-than-optimum blood glucose concentration: Comparative risk assessment. Lancet.

[33] Kaneko H, Itoh H, Kiriyama H, Kamon T, Fujiu K, Morita K, Michihata N, Jo T, Takeda N, Morita H (2021). Fasting plasma glucose and subsequent cardiovascular disease among young adults: Analysis of a nationwide epidemiological database. Atherosclerosis.

[34] Volpe M, Borghi C, Perin P, Chiariello M, Manzato E, Miccoli R, Modena M, Riccardi G, Sesti G, Tiengo A (2010). Cardiovascular Prevention in Subjects with Impaired Fasting Glucose or Impaired Glucose Tolerance. High Blood Pressure & Cardiovascular Prevention.

[35] Liu S: Study on the incidence of prediabetes and diabetes and related factors based on a cohort population from 10 provinces in China. Master. Chinese Center for Disease Control and Prevention 2020.

[36] Zhang X, Zhao X, Huo L, Yuan N, Sun J, Du J, Nan M, Ji L: Risk prediction model of gestational diabetes mellitus based on nomogram in a Chinese population cohort study. Scientific Reports 2020, 10. 10.1038/s41598-020-78164-x PMC7718223 33277541

[37] James J: Personalised medicine, disease prevention, and the inverse care law: More harm than benefit? European journal of epidemiology 2014, 29. 10.1007/s10654-014-9898-z 24729105

[38] Kraegen E, James D, Jenkins A, Chisholm D (1985). Dose–response curves for in vivo insulin sensitivity in individual tissues in rats. The American journal of physiology.

[39] Andrews R, Greenhaff P, Curtis S, Perry A, Cowley AJ (1998). The effect of dietary creatine supplementation on skeletal muscle metabolism in congestive heart failure. European heart journal.

[40] Harita N, Hayashi T, Sato K, Nakamura Y, Yoneda T, Endo G, Kambe H (2008). Lower Serum Creatinine Is a New Risk Factor of Type 2 Diabetes. Diabetes care.

[41] Zierath JR, Krook A, Wallberg-Henriksson H (2000). Insulin action and insulin resistance in human skeletal muscle. Diabetologia.

[42] Yamada T, Fukatsu M, Suzuki S, Wada T, Joh T (2011). Elevated serum uric acid predicts impaired fasting glucose and type 2 diabetes only among Japanese women undergoing health checkups. Diabetes & Metabolism.

[43] Miyake T, Kumagi T, Furukawa S, Hirooka M, Kawasaki K, Koizumi M, Todo Y, Yamamoto S, Abe M, Kitai K (2014). Hyperuricemia Is a Risk Factor for the Onset of Impaired Fasting Glucose in Men with a High Plasma Glucose Level: A Community-Based Study. PloS one.

[44] Nakanishi N, Okamoto M, Yoshida H, Matsuo Y, Suzuki K, Tatara K (2003). Serum uric acid and risk for development of hypertension and impaired fasting glucose or Type II diabetes in Japanese male office workers. European journal of epidemiology.

[45] Taniguchi Y, Hayashi T, Tsumura K, Endo G, Fujii S, Okada K (2001). Serum uric acid and the risk for hypertension and Type 2 diabetes in Japanese men: The Osaka Health Survey. Journal of hypertension.

[46] Alfredo Q, Natali A, Baldi S, Frascerra S, Sanna G, Ciociaro D, Ferrannini E (1995). Effect of insulin on uric acid excretion in humans. The American journal of physiology.

[47] Khosla U, Zharikov S, Finch J, Nakagawa T, Roncal C, Krotova K, Block E, Prabhakar S, Johnson R (2005). Hyperuricemia induces endothelial dysfunction. Kidney international.

[48] Yang X, Xu C, Wang Y, Cao C, Tao Q, Zhan S, Sun F: Risk prediction model of dyslipidaemia over a 5-year period based on the Taiwan MJ health check-up longitudinal database. Lipids in Health and Disease 2018, 17. 10.1186/s12944-018-0906-2 PMC6240269 30447693

